# Quantum light drives electrons strongly at metal needle tips

**DOI:** 10.1038/s41567-025-03087-1

**Published:** 2025-11-07

**Authors:** Jonas Heimerl, Andrei Rasputnyi, Jonathan Pölloth, Stefan Meier, Maria Chekhova, Peter Hommelhoff

**Affiliations:** 1 Department of Physics, Friedrich-Alexander-Universität Erlangen-Nürnberg, Erlangen, Germany; 2 Max Planck Institute for the Science of Light, Erlangen, Germany; 3 Faculty of Electrical and Computer Engineering, Technion-Israel Institute of Technology, Haifa, Israel; 4 Faculty of Physics, Ludwig-Maximilians-Universität München, Munich, Germany

**Keywords:** Single photons and quantum effects, Surfaces, interfaces and thin films, Matter waves and particle beams, Attosecond science

## Abstract

Attosecond science relies on driving photoemitted electrons with the strong optical field of a laser pulse, which represents an intense classical coherent state of light. Bright squeezed vacuum is a quantum state of light that is also intense enough to drive strong-field physics. However, its mean optical electric field is zero, suggesting that, in a semi-classical view, electrons should not experience strong driving. The question arises if and how this quantum state of light can generate signatures of attosecond dynamics in strong-field photoemission. Here we show that the key signatures of strong-field physics—the high energy plateau and subsequent cut-off—also appear under driving of a needle tip by bright squeezed vacuum, but only when we post-select electron energy spectra on the individual photon number of each pulse. When averaging over many shots, we observe broad energy spectra without a plateau. This suggests that electrons driven by bright squeezed vacuum behave as if driven by an ensemble of coherent states of light. Our findings bridge strong-field physics and quantum optics, offering insights into bright squeezed vacuum and other quantum light states, and suggest the use of strongly driven electrons as quantum light sensors.

## Abstract (web summary)

The common description of strong-field light–matter interaction neglects the quantum-optical nature of the driving field. Now signatures of strong-field photoemission appear in electron energy spectra when driving with non-classical light.

## Main

Investigating how intense ultrashort light pulses interact with matter is at the heart of strong-field and attosecond physics. Over 40 years of research have led to an unprecedented understanding of how electrons are driven by a strong optical field on the attosecond timescale and subnanometre length scale at atoms^1–3^, and more recently, also at metal surfaces^4–10^. These insights have led to an understanding of electron dynamics on their natural timescales, achieving stunning precision down to single-digit attoseconds in atoms, molecules and solids, and at solid surfaces^11–15^.

According to the three-step model of strong-field physics, electrons tunnel-emitted into an intense optical field can be driven back to the parent matter^16^. There, they may recombine and emit high-harmonic radiation or rescatter elastically to subsequently gain higher energy. This results in the famous plateau, either in the high harmonic spectrum or in the energy spectrum of the electrons^17,18^. This all rests on the assumption that the driving field is a classical sine-wave-like optical field, well justified by the large number of photons involved, rendering quantum fluctuations in the amplitude and phase of the light negligible: already for a small pulse energy of 1 nJ the number of involved photons for a near-infrared driving field is of the order 10^10^, leading to intensity fluctuations of ~0.001% according to the Poissonian photon statistics of coherent light. For this reason, the common description of the strong-field light–matter interaction neglects the quantum-optical nature of the driving field.

Two different approaches have recently been proposed to search for quantum optical signatures in strong-field processes, that is, to explore strong-field quantum optics^19–21^. In one approach, classical coherent light is used as the driver for strong-field processes that generate non-classical states^22–24^. Initial experiments along this line show the generation of non-classical states at the wavelength of the driver by conditioning on the high harmonic generation, maintaining the classical intuition of the electron dynamics^25,26^. Recent studies show quantum correlations between non-perturbative harmonics^27^ and predict non-Gaussian states of harmonics and entanglement between harmonics^28^.

**Fig. 1:**
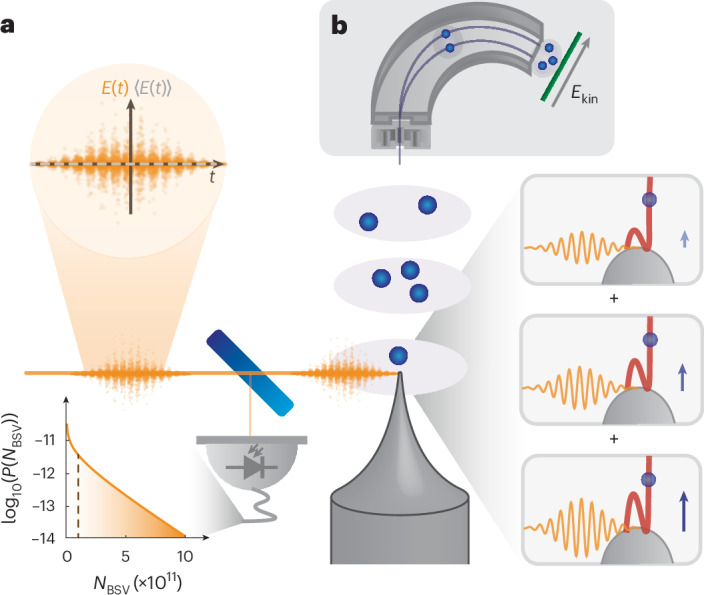
Set-up for the measurement of strong-field electron energy spectra driven by quantum light. **a**, BSV has a mean electric field of zero (〈**E**〉 = 0, dashed grey line) with a large variance oscillating at twice the carrier frequency (circled inset). Ninety-six per cent of the BSV are sent to the tungsten needle held in an ultrahigh vacuum chamber (not shown), while the photon number of each shot is monitored with the 4% BSV pick-up at a photodiode (grey, losses not included). The calculated photon number distribution *P*(*N*_BSV_) for a mean of 〈*N*_BSV_〉 = 10^11^ photons per pulse (dashed brown line) is shown on the bottom left. Electrons emitted in a nonlinear photoemission process are driven in the strong optical near field and rescatter at the tip surface (see boxes), generating high-energy electrons. We discuss below why the insets show classical coherent light states. **b**, A sketch of the home-built shot-resolving electrostatic low-energy electron spectrometer. We use an electrostatic quadrupole lens in front of the deflector to focus the electron beam emitted from the tip into the spectrometer. The energy of each electron *E*_kin_ is recorded with the help of a microchannel plate and phosphor screen placed in the detector plane of the spectrometer (green line).

The second approach towards strong-field quantum optics, which we focus on here, is to directly resort to a non-classical driving field of light. We choose bright squeezed vacuum (BSV)^29^, an intriguing light state whose mean electric field 〈**E**〉 is zero at all times. Its variance oscillates at twice the carrier frequency (Fig. 1a), and it can still represent intense light because its mean intensity *I* ∝ 〈**E**^2^〉 is proportional to the electric field squared^30^, and $I\propto {\sinh }^{2}r$, where the squeezing parameter *r* can be as high as 15 (refs. ^31,32^). Notably, the intuitive semi-classical picture of strong-field dynamics fails dramatically, as no acceleration of an electron would be classically expected because 〈**E**〉 = 0. Clearly, new theoretical frameworks are needed to take the quantum nature of the light field into account, several of which were recently developed^22,23,28,33–38^.

Initial experiments using non-classical driving light demonstrated that the photon number statistics of BSV are transferred to the number statistics of photoemitted electrons^39^, and, in a two-colour experiment where one colour is in a non-classical state, also to the statistics of high harmonic orders^40,41^. Furthermore, BSV was used to generate high harmonics in solids^32^. Most recently, a two-colour gas-phase high-harmonic generation (HHG) experiment showed in situ quantum state tomography^41^.

Yet, thus far, neither the rescattering of electrons nor the plateau in HHG driven by non-classical light has been demonstrated in experiments. Clearly, such measurements are crucial for verifying current theory models and for investigating how the system of electrons and photons is potentially entangled. Intriguingly, based on rescattering, the subcycle properties of quantum states of light can be probed with attosecond resolution. In addition, such experiments can shed light on the fundamental and non-trivial questions of quantum measurements at extremely short timescales.

## Experiment

In the experiment, we generate temporally and spatially single-mode BSV from an unseeded optical parametric amplifier (see ref. ^32^ for details and Fig. 1a for the set-up). The BSV is centred at 1,600 nm (photon energy of 0.77 eV) and has a pulse duration of 25 fs. The repetition rate of the pump laser is 1 kHz. The available pulse energy of BSV is on the order of several hundred nanojoules. We adjust the mean pulse energy for the experiment by varying the pulse energy of the optical parametric amplifier pump. Furthermore, we pick off a small percentage of each BSV pulse by a fused silica window reflection to monitor the pulse-to-pulse photon number fluctuations using a photodiode. The measured photon number at the photodiode *N*_BSV_ is proportional to the number of photons in each BSV pulse.

The BSV pulses are sent into an ultrahigh vacuum chamber with a base pressure of *p* < 1 × 10^−8^ hPa, where they are focused to 8 μm (1/e^2^ intensity radius) using an off-axis parabolic mirror. A metal needle tip with a radius of a few tens of nanometres is situated on a three-axis nanopositioner, with which we align the tip apex to the optical focus. Uncompensated group velocity dispersion leads to temporal broadening of the light pulses to ~38 fs at the tip (still single mode). The electrons are emitted in a nonlinear photoemission process, where the nonlinearity is given mainly by the ratio of the workfunction of tungsten (~5 eV) and the photon energy^39,42^. After the photoemission from the negatively biased tip (*V* = −310 V), the electrons travel towards a home-built low-energy spectrometer, which measures both the number of electrons (up to a few tens per shot) and each electron’s energy for each individual laser pulse with an energy resolution of ~2 eV (Fig. 1b). The electron spectrometer is based on an electrostatic cylindrical deflector analyser^43^. A microchannel plate equipped with a phosphor screen is used to image individual electrons with a camera. This camera and the photodiode in front of the vacuum chamber are synchronized to the repetition rate of the laser, allowing us to correlate the photon number *N*_BSV_ and the number and energy of the electrons for each light pulse.

## Shot-averaged strong-field spectra

**Fig. 2:**
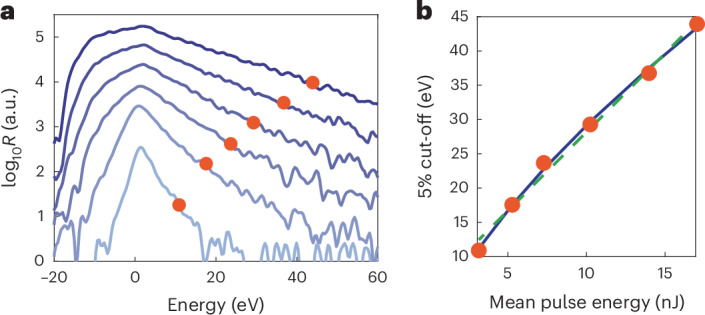
Measured shot-averaged electron energy spectra. **a**, Electron energy spectra driven by BSV with increasing mean pulse energy (from light blue to dark blue: [3.2, 5.3, 7.3, 10.2, 14.0, 17.0] nJ). For better visibility, the spectra are shifted vertically (the shift of consecutive spectra is 0.4 on the logarithmic axis). The energy offset resulting from the d.c. bias voltage is subtracted. The red dots mark the points where the count rate *R* has dropped to 5% of each maximum, defining the 5% cut-off. **b**, The 5% cut-off position as a function of mean pulse energy from **a**. The dashed green line is a linear fit, and the blue curve is a power-law fit. The best fit exponent of the latter is 0.65 ± 0.2. Source data

Figure 2a shows electron energy spectra for a range of mean pulse energies of BSV from 3.2 nJ to 17.0 nJ. For each electron spectrum at a fixed BSV mean power, we record 10,000 images accumulating electrons from ~13 laser pulses per image, that is, each spectrum sums up electrons from a total of 1.3 × 10^5^ BSV pulses. For increasing pulse energy (light to dark blue) the energy spectra clearly broaden, and the slope around 10… 40 eV becomes less steep.

**Extended Data Fig. 1:**
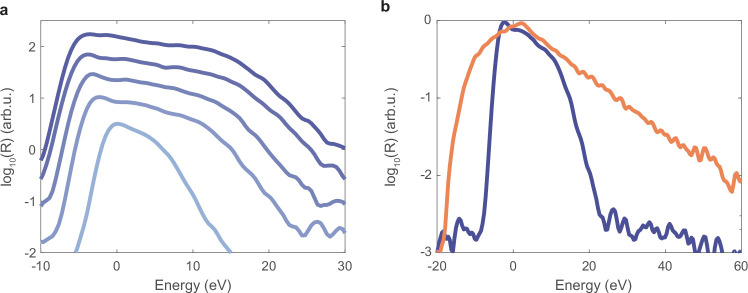
Experimental strong-field spectra from coherent light. **a**, Strong-field spectra measured with coherent light at a central wavelength of 1600 nm. For better visibility, the spectra are shifted on the vertical axis. See text for details. **b**, Comparison of coherent state-driven (blue) and BSV-driven (orange) electron spectra for comparable mean peak intensities. Clearly, the BSV spectrum extends towards much higher energies, as explained in the main text. Source data

Notably, we observe electron energies exceeding 60 eV, where the energy range of the spectrometer ends. For the highest mean pulse energy, we would expect a classical cut-off energy of 10 eV based on the 10-*U*_p_ law^8,18^. Thus, within the dynamic range of our system, electrons driven by coherent light are expected to have energies no greater than approximately 18 eV (beyond the 10-*U*_p_ cut-off; Extended Data Fig. 1). The observed energies for BSV exceed this value substantially; however, we observe neither a plateau nor a cut-off.

To quantify the scaling of the high energy electrons with the mean BSV pulse energy, we define the cut-off for these spectra to lie at 5% of the maximum rate. For increasing pulse energy, this 5% cut-off position shifts to larger values in a slightly sublinear manner (Fig. 2b). Using a power-law fit to the data (blue), we retrieve an exponent of 0.65 ± 0.2. Similar to simulations around HHG with BSV driving, we observe an exponent below 1 (ref. ^34^). We note that the exact scaling depends on the chosen threshold and also varies slightly from tip to tip.

## Shot-resolved strong-field spectra

By measuring individual electron spectra for each light pulse (Methods), we can now gain insight into how the averaged spectra are formed. Even more importantly, we will conceptually understand how the interaction of BSV and matter in the strong-field regime can be interpreted based on correlating spectra and photon number statistics. Quantum-optically speaking, we will post-select spectra based on the photon number.

**Fig. 3:**
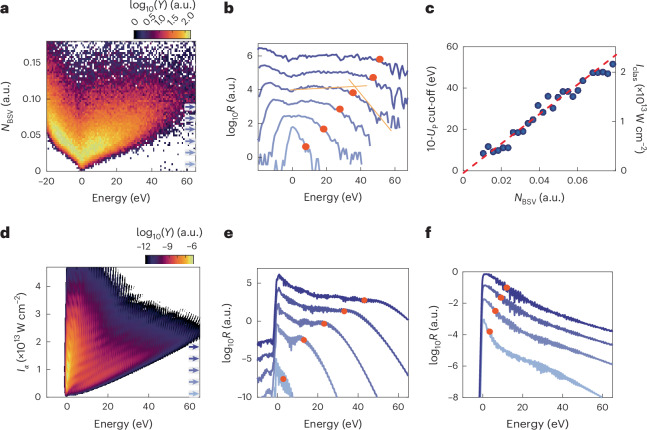
Measured and simulated shot-resolved energy spectra. **a**, Map of the measured electron energy spectra for a fixed mean BSV pulse energy of 21 nJ. The horizontal axis shows the energy of each detected electron and the vertical axis the BSV photon number *N*_BSV_ measured at the photodiode (Fig. 1a). The colour encodes the yield *Y*. For increasing *N*_BSV_, the energy spectra broaden substantially, from close to the minimum width of 2 eV up to the maximum detectable electron energy of 65 eV. **b**, Six line-out spectra from **a**. The positions of the line-outs are indicated by blue arrows in **a**. The shape of these line-out spectra resembles that of well-known strong-driving spectra—with classical coherent laser light: they clearly show the plateau and the 10-*U*_p_ cut-off (red dots). **c**, Cut-off positions from all line-outs in **a** as a function of the photon number *N*_BSV_. The right-hand axis indicates the expected intensity *I*_clas_ for coherent light calculated back from the 10-*U*_p_ law (see ‘Shot-resolved strong-field spectra’). The dashed red line is a linear fit to the data, indicating a scaling behaviour identical to coherent driving. **d**, Simulated shot-resolved electron energy spectra from integrating the TDSE. The vertical axis shows the intensity of a coherent driver *I*_*α*_. The shape matches the experimentally obtained one well, and we stress that these numerical results are obtained with a classical driving field. The fast oscillations are a result of inter- and intracycle effects^7,53^, not visible in the experiment. We note further that no electrons appear at negative energies because the simulation is based on single-electron dynamics (Methods). Extended Data Fig. 3 contains a version of this figure with more detailed and additional discussions. **e**, Line-out energy spectra from **d** for increasing intensities of coherent light ([0.12, 0.54, 0.96, 1.38, 1.8] × 10^13^ W cm^−^^2^; light blue to dark blue and indicated by blue arrows in **d**). Red circles indicate the calculated cut-off positions. **f**, Simulated electron energy spectra for BSV with four mean intensities of [1.2, 2.5, 3.8, 5.0] × 10^12^ W cm^−2^, equivalent to the classical cut-off positions [3.1, 6.1, 9.0, 12.0] eV (indicated by red circles). Source data

We set the mean pulse energy to 21 nJ and record 6 × 10^5^ spectra with an average of 0.22 electrons detected per BSV pulse. Electron energy spectra versus the BSV photon number *N*_BSV_ are shown in Fig. 3a. Clearly, the electron energy spectra broaden substantially with increasing photon number and exhibit a clear correlation between the width of the spectrum and the number of photons detected in the driving BSV pulse. A dominant broadening is observed towards positive energies, with a smaller broadening towards negative energies. The broadening to negative energies is due to Coulomb repulsion of the electrons. Simulations confirm that Coulomb repulsion effects can be neglected for the large positive energies (see Extended Data Fig. 4 and the corresponding discussion in the Methods).

Line-outs (shown in Fig. 3b) of the data in Fig. 3a more clearly highlight the crucial results shown in the spectra–photon number correlation plot (post-selected spectra). Intriguingly, the shape of these line-outs resembles those of strong-field spectra well known for coherent driving: a clearly discernible plateau ending in a clearly visible cut-off, which increases with the photon number (see the Methods or, for example, ref. ^7^). This is a clear indication that the post-selection on the shot’s photon number acts like a projection of the driving BSV on the amplitude of a coherent state, for which the semi-classical picture of strong-field physics holds. From this we infer that the clearly visible cut-off is the famous 10-*U*_p_ cut-off.

To gain quantitative insights, we use two linear functions in the semi-logarithmic representation to extract the 10-*U*_p_-cut-off position for each line-out (red circles), and all BSV bins of Fig. 3a (ref. ^44^). The retrieved 10-*U*_p_-cut-off positions are shown in Fig. 3c. Identical to coherent driving light, the cut-off position increases proportionally to the post-selected photon number, as shown by a linear fit to the data (red line). We note in passing that the broad photon-number distribution of BSV in conjunction with post-selection allows us to monitor the electron dynamics without scanning the driving field amplitude.

## Discussion

The direct connection between photon number and clearly visible 10-*U*_p_ cut-off allows us to address three important questions: (1) What determines the shape of the shot-averaged spectra? (2) What is the intensity of the non-classical light averaged over just one optical cycle as experienced by the driven electron? And (3) does optical field enhancement at the tip apex arise for such a non-classical light field, like for coherent light?

Question 1 is answered by constructing shot-averaged spectra from the shot-resolved spectra. Adapting pioneering theory work^34^ to electron spectra from metal needle tips, we can obtain an averaged electron spectrum for a fixed mean BSV intensity 〈*I*_BSV_〉 as the sum of individual shot-resolved, that is, post-selected, electron spectra weighted by the likelihood for this shot’s intensity to arise, reflected by the Husimi function of BSV. The Husimi function of BSV is a quasi-one-dimensional distribution *Q*_BSV_(*α*) ≈ *Q*_BSV_(∣*α*∣)*δ*(*ϕ*_*α*_), where *α* is the complex amplitude of the electric field and *ϕ*_*α*_ its phase (Methods). The probability distribution of the electric field amplitudes *Q*_BSV_(∣*α*∣) can be rewritten as function of the (coherent driver) intensity *I*_*α*_ (ref. ^34^), which is equivalent to the expression for the BSV photon number distribution^45^:

1 $${Q}_{{\rm{BSV}}}({I}_{\alpha },\langle {I}_{{\rm{BSV}}}\rangle )=\frac{1}{\sqrt{2\uppi \langle {I}_{{\rm{BSV}}}\rangle {I}_{\alpha }}}{{\mathrm{e}}}^{-{I}_{\alpha }/(2\langle {I}_{{\rm{BSV}}}\rangle )}.$$

The shot-averaged electron energy spectrum *S*_BSV_(*E*) for BSV driving is then given by

2 $${S}_{{\rm{BSV}}}(E,\langle {I}_{{\rm{BSV}}}\rangle )=\int{S}_{{\rm{coh}}}(E,{I}_{\alpha }){Q}_{{\rm{BSV}}}({I}_{\alpha },\langle {I}_{{\rm{BSV}}}\rangle ){\rm{d}}{I}_{\alpha },$$

where *S*_coh_(*E*, *I*_*α*_) is the electron spectrum resulting from driving with classical coherent light with intensity *I*_*α*_ and *E* is the electron energy (Methods).

**Extended Data Fig. 2:**
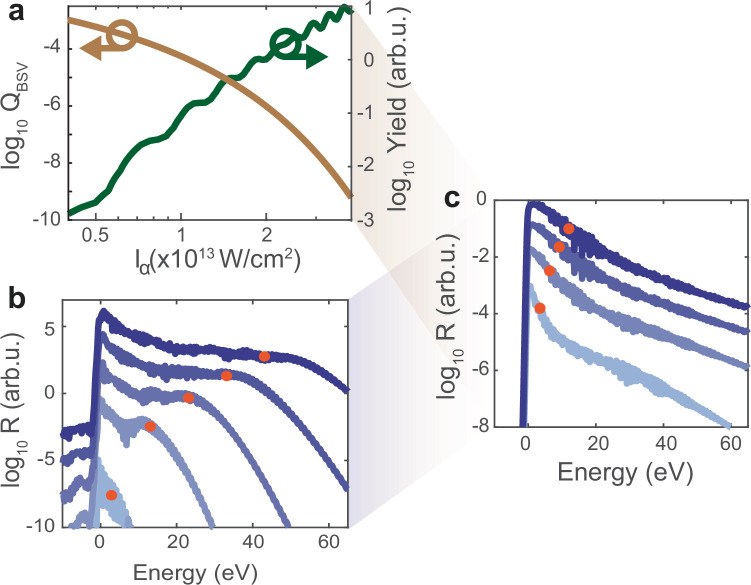
Husimi function and simulated shot-averaged electron energy spectra. **a**, Calculated amplitude part of the Husimi function *Q*_BSV_ (brown, left-hand axis) and simulated electron yield (green, right-hand axis) as function of peak intensity *I*_*α*_, shown on a double-logarithmic scale. **b**, Simulated CEP-averaged electron energy spectra for increasing intensities of coherent light ([0.12, 0.54, 0.96, 1.38, 1.8] × 10^13^ W/cm^2^; light blue to dark blue). Red circles indicate the calculated cut-off positions. **c**, Simulated electron energy spectra for BSV with four mean intensities of [1.2, 2.5, 3.8, 5.0] × 10^12^ W/cm^2^, equivalent to the classical cut-off positions [3.1, 6.1, 9.0, 12.0] eV (indicated by red circles). Panels **b** and **c** are identical to Fig. 3e,f and are shown here for clarity. Source data

Based on the integration of the time-dependent Schrödinger equation (TDSE; Methods), we simulate energy spectra for various coherent light intensities (Fig. 3d,e) and calculate the spectra expected for BSV for four different mean intensities of [1.2, 2.5, 3.8, 5.0] × 10^12^ W cm^−2^ from equation (2) (Fig. 3f). Clearly and like the experimental shot-averaged spectra (Fig. 2a), the resulting simulated spectra feature no prominent plateaus, in contrast to Fig. 3e. Further, the sum (equation (2)) leads to energies that exceed the classically expected cut-off energies (10-*U*_p_ law, indicated by red dots) by multiples. These shot-averaged spectra, given by the incoherent sum, agree qualitatively and quantitatively with the measured data. They directly explain the presence of high-energy electrons as well as the disappearance of the plateau—both resulting from the interplay between BSV’s large intensity fluctuations, particularly intensity outliers, and the nonlinear electron emission yield (Extended Data Fig. 2). Hence, the theory developed by Gorlach et al.^34^ fully aligns with our experimental observation, demonstrating that a classical interpretation of the optical electric field of BSV is no longer possible.

To address question 2, we consider the driven electrons local probe particles and attain the optical intensities responsible for driving the electrons strongly from the observed cut-off, which we identified as 10-*U*_p_ cut-off of coherent driving light. We obtain what we call the classical intensity *I*_clas_ = *E*_obs-cutoff_ × (*cϵ*_0_*m*)/(2*e*^2^) × (4*ω*^2^/10.007), where *c* is the speed of light, *ϵ*_0_ is the vacuum permittivity, *e* and *m* are the electron charge and mass, *ω* is the driving light’s angular frequency and *E*_obs-cutoff_ is the cut-off energy shown in Fig. 3b (red spheres). *I*_clas_ is shown on the right-hand axis of Fig. 3c. It corresponds to the peak intensity of the strongest optical BSV cycle and includes any potential field enhancement arising at the nanometric needle tip. Hence, here we use the strongly driven electron as a sensor for the optical electric field of the non-classical light, on a subcycle timescale^44^ (see discussion on pseudo-coherent state below).

**Extended Data Fig. 3:**
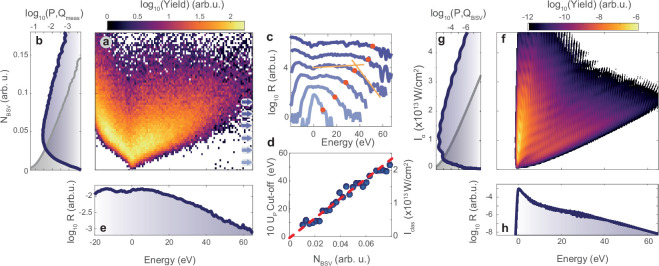
Measured and simulated shot-resolved energy spectra. Extended version of Fig. 3. **a**, Map of the measured electron energy spectra for a fixed mean BSV pulse energy of 21 nJ. The horizontal axis shows the energy of each detected electron and the vertical axis the normalized BSV photon number *N*_BSV_ measured at the photodiode. For increasing *N*_BSV_, the energy spectra broaden substantially, from close to the minimum width of 2 eV up to the maximum detectable electron energy of 65 eV. **b**, Normalized yield *P* of electrons as a function of *N*_BSV_ (blue, marginal distribution of panel **a** onto vertical axis). This curve is the product of the BSV intensity probability distribution and the total ionization yield (see Extended Data Fig. 2a and Extended Data Fig. 2). The gray line is the measured photon number distribution. **c**, Six line-out spectra from **a**. These spectra clearly show the plateau and the 10-*U*_p_-cut-off (red dots) as known from coherent driving light. The arrows in **a** indicate the position of the line out. **d**, Cut-off positions from all line-outs in **a** as a function of the photon number *N*_BSV_. The right-hand axis indicates the expected intensity *I*_clas_ for coherent light calculated back from the 10-*U*_p_-law. The red dashed line is a linear fit to the data, indicating also the identical scaling behavior as for coherent driving. **e**, Shot-averaged energy spectrum obtained by the projection of **a** on the horizontal axis, matching the shot-averaged spectrum in Fig. 2a. **f**, Simulated shot-resolved electron energy spectra from integrating the time-dependent Schrödinger equation and weighting each intensity by the Husimi function (Eq. (2)). The vertical axis shows the intensity of a coherent driver *I*_*α*_. **g,h**, Projections onto the respective axes as in **b,e**. Source data

Furthermore, we obtain the mean BSV intensity experienced by the driven electrons 〈*I*_BSV_〉, that is, including any potential field enhancement, as $\langle {I}_{{\rm{BSV}}}\rangle ={\sum }_{{N}_{{\rm{BSV}}}}{Q}_{{\rm{meas}}}({N}_{{\rm{BSV}}})\times {I}_{{\rm{clas}}}({N}_{{\rm{BSV}}})=3.9\times 1{0}^{12}\,{\rm{W}}\,{{\rm{cm}}}^{-2}$ for the data in Fig. 3, where the measured Husimi function *Q*_meas_ coincides with the photon-number distribution detected at the photodiode (Extended Data Fig. 3). This intensity arises from electron dynamics occurring within a suboptical cycle duration—otherwise, the spectra would not resemble those generated by coherently driven electrons.

Question 3 can now be directly addressed: the ratio of the intensity experienced by the electron to the intensity in the bare focus—determined from the experimental parameters above—yields the square of the field enhancement factor^44^. This field enhancement factor, well known for coherent light pulses, is a result of charge accumulation at the tip surface as the tip radius is much smaller than the incident wavelength. It is defined as FE = ∣*E*_nf._/*E*_inc._∣, which is the ratio of the optical near field at the tip surface and the incident electric field. Here, we attain the field enhancement factor from the slope in Fig. 3c as FE = 3.4 ± 0.6. This value agrees with field enhancement factors expected for coherent light and tips of similar size^46^. Hence, it appears that the cut-off energies are proper markers for the peak intensity present at the tip apex and that optical field enhancement also arises for the highly fluctuating BSV, like for coherent light. This no longer holds when the build-up time of the field enhancement—that is, the electron response within the tip apex, as determined by the plasma frequency—exceeds the mean fluctuation time of the driving light.

It is essential to realize that we have arrived at a point where we decompose the BSV driving field into a set of classical fields. This is a direct consequence of the measurement procedure based on the post-selection on the particular photon number of the driving field. This post-selection can be seen as a projection onto a pseudo-coherent state, that is, a coherent state with undefined phase^47^. In this way, a non-zero optical electric field amplitude arises, allowing us to infer the optical-field statistics from the cut-off energy of the electron generated by the strong light–matter interaction. Our results do not seem to display contributions of the quantum coherence of the driving light, that is, that BSV is a coherent superposition of multiple coherent states^48,49^. A natural continuation of our work is to add a classical coherent light field at doubled frequency to the BSV field, in analogy to what was shown in refs. ^40,41^, but now with reversed roles. Metal needle tips are an ideal tool to investigate what happens when BSV is the main driver and not only a small perturbation. In such a two-colour experiment, the particular quadrature of the BSV field, either squeezed or antisqueezed depending on the phase between two field components, will be highlighted for the photo-electron emission, and the electric field amplitude fluctuations can be recovered from the energy-resolved statistics of direct and rescattered electrons. In this way, the additional field will allow us not only to reconstruct modified electron trajectories but also to perform quantum state tomography of the BSV field^40,41^. The novel techniques of photo-electron quantum state tomography^50^ and time-domain tomography of light^51,52^ will also be valuable tools for accessing the quantum coherence of electrons strongly driven by BSV.

## Methods

### Bright squeezed vacuum

The experimental set-up for the generation of single-mode BSV is described in ref. ^32^. The measured second-order correlation function of the light in our experiments is *g*^(2)^ > 2.8, which shows the nearly single-mode character of the generated light. We calculate this value from *Q*_meas_ in Extended Data Fig. 3b (grey curve).

In equations (1) and (2), we exploited the fact that the Husimi function of BSV can be simplified to a one-dimensional expression as a function of the intensity. More generally, the Husimi function of a squeezed state is given by

3 $${Q}_{{\rm{BSV}}}(\alpha =x+iy)=\frac{1}{\uppi \cosh (r)}\exp \left(-\frac{2{y}^{2}}{1+{{\mathrm{e}}}^{-2r}}-\frac{2{x}^{2}}{1+{{\mathrm{e}}}^{2r}}\right),$$

where *r* is the squeezing parameter^34^. For strong squeezing, like in our case, the distribution is heavily stretched along the antisqueezed quadrature and is close to a delta function along the squeezed one. As shown in ref. ^34^, one can then approximate the Husimi function by

4 $${Q}_{{\rm{BSV}}}({{\mathcal{E}}}_{\alpha })=\frac{1}{2\uppi | \bar{{\mathcal{E}}}{| }^{2}}\exp \left(-\frac{| {{\mathcal{E}}}_{\alpha }{| }^{2}}{2| \bar{{\mathcal{E}}}{| }^{2}}\right)\delta ({\phi }_{\alpha }),$$

where ${{\mathcal{E}}}_{\alpha }\propto \alpha$ is the complex electric field and $\bar{{\mathcal{E}}}$ is the mean electric field. As the intensity is ${I}_{\alpha }\propto | {{\mathcal{E}}}_{\alpha }{| }^{2}$, we end up with equation (1), where the additional factor $1/\sqrt{{I}_{\alpha }}$ comes from normalization.

### Experimental shot-resolved electron energy spectra

Due to the design of our electron energy spectrometer (see main text), the energy range we can resolve depends on the bias voltage applied to the metal needle tip and the potentials applied to the electron deflector. For a bias voltage of −310 V, we can measure up to 60 eV energy gain of the electrons. We can extend this range by two consecutive measurements with two different bias voltages of the tip. This was done for the shot-resolved measurement in Fig. 3, with bias voltages − 270 V and − 310 V. Through post-processing, we combine these two measurements to obtain shot-resolved energy spectra with an extended energy range.

### Experimental strong-field spectra from coherent light

For comparison with the BSV case, we measured averaged strong-field spectra with coherent light pulses at the same wavelength of 1,600 nm. In this case, the pulse duration was *τ* = 70 fs. In Extended Data Fig. 1a we show spectra for pulse energies of [24, 33, 40, 45, 51] nJ. We observe a clear plateau for all pulse energies. Furthermore, the maxima of the spectra shift towards negative energies at higher pulse energies, similar to our BSV measurement shown in Fig. 3a. For the highest pulse energy, where the near-field intensity is *I*_coh_ = 6.3 × 10^12^ W cm^−2^, the shift is 3.5 eV. From this, we infer that the observed shift of the maxima in the case of BSV driving (Fig. 3b) is not related to the quantum state of the driving light, but rather results from classical Coulomb repulsion among the emitted electrons (see the discussion on multi-electron effects and simulations below).

Furthermore, we show in Extended Data Fig. 1b the comparison of a spectrum recorded with coherent light (blue) and one recorded with BSV (orange). Here, we set the pulse energies of *E*_P,coh_ = 33 nJ and *E*_P,BSV_ = 14 nJ such that the classically calculated mean intensities are similar, despite the different pulse durations. As explained before, the spectrum generated from BSV extends over a much broader range compared with the coherent case.

### Simulation of coherent-light strong-field spectra

We calculate the energy spectra of the electrons by solving the single-electron TDSE for a particle in a box. Past experiments have shown that the assumption of a single bound state can explain the relevant physics also for metallic needle tips^15^. The high nonlinearity leads to an energetically narrow emission of the electrons, which makes it possible to use only one bound state (see ref. ^54^). To account for the symmetry breaking of the metal–vacuum interface, the bound state sees the electric field of the laser pulse only from one side. Consequently, we are also only interested in the wavefunction that is emitted into the vacuum. For details of the implementation and code examples, we refer to ref. ^15^. Here, we assume a workfunction (ionization potential) of 6 eV. Furthermore, we include a relatively strong static electric field of −0.5 V nm^−1^, which is present in the experiment due to the bias voltage applied and the small tip size. For computational reasons, we assume a pulse duration of *τ* = 25 fs instead of 38 fs, because the grid size in the simulation scales approximately quadratically with pulse duration. Both pulse durations represent multi-cycle laser pulses, where the exact pulse duration has no strong effect on the spectral shape. Furthermore, we include the position dependence of the optical near field $\xi (x)=1+({\rm{FE}}-1)\times \exp (-x/\zeta )$, where FE is the field enhancement factor at the surface of the tip, *x* is the distance from the tip apex and *ζ* is the decay length^44,55,56^. Here, we assume a near-field decay length of *ζ* = 20 nm and a FE of 3, corresponding to the expected parameters for a tip with a radius of a few tens of nanometres^46^.

We simulate the energy spectra for intensities from 1 × 10^11^ W cm^−2^ to 5 × 10^13^ W cm^−2^ and two CEPs of *ϕ*_CEP_ = [0, 1] ⋅ π. The spectra for each intensity shown in the main text are CEP-averaged due to a non-stabilized CEP in the experiment.

We calculate the yield for a fixed intensity by the sum of all energies larger than zero. The simulated yield as a function of intensity exhibits, in addition to the overall increase, a periodic modulation (Extended Data Fig. 2a). This modulation is due to channel closing.

As is well known from previous works^8^, the optical near field can lead to final electron energies smaller than those expected from the 10-*U*_p_ law due to a quenched quiver motion. Here, the tip radius (near-field decay) is too large to observe such an effect strongly^55^, which is why we still assume a 10-*U*_p_ law in both simulation and experiment (see also further discussions below). Small deviations from this scaling would only result in a scaling factor for the intensities shown in Fig. 3, without affecting our interpretation.

### Simulation of shot-averaged spectra driven by BSV

In Extended Data Fig. 2a, we show the amplitude part of the Husimi function of BSV, *Q*_BSV_(∣*α*∣) (brown curve), calculated for a mean intensity of 〈*I*_BSV_〉 = 1.5 × 10^12^ W cm^−2^ and, in the same plot, the simulated total yield for coherent driving (green, right axis) as a function of the intensity *I*_*α*_ (see ‘Simulation of coherent-light strong-field spectra’ for details of the simulation). Clearly, lower intensities (below ~1 × 10^13^ W cm^−2^) have a high probability of occurring according to the Husimi function but lead to a small electron yield. Therefore, this contribution to the count rate *R* in the electron spectrum competes with that from high intensities, which show a higher electron yield but appear less often.

Based on the integration of the TDSE, we simulate energy spectra for various coherent light intensities (light blue to dark blue; Extended Data Fig. 2b) and calculate the spectra expected for BSV for four different mean intensities of [1.2, 2.5, 3.8, 5.0] × 10^12^ W cm^−2^ from equation (2) (Extended Data Fig. 2c). Clearly, and similar to the experimental results shown in Fig. 2a, the resulting spectra feature no prominent plateaus, in contrast to Extended Data Fig. 2b. Furthermore, the sum leads to energies that exceed the classically expected cut-off energies (10-*U*_p_ law, indicated by red dots) by multiples. These shot-averaged spectra agree qualitatively with the measured ones and directly explain both the observed high-energy electrons and the washing out of the plateau. Both effects originate from the interplay between BSV’s large intensity fluctuations—especially intensity outliers—and the nonlinear electron emission yield.

### Detailed analysis of shot-resolved strong-field spectra driven by BSV

Here, we show more details on the experimental and simulated shot-resolved spectra together with a detailed version of Fig. 3, shown in Extended Data Fig. 3. The extended version shows the marginals of the correlation maps, which are the shot-average spectra (Extended Data Fig. 3e,h) and the probability of measuring an electron event *P* as a function of the photon number, or intensity (Extended Data Fig. 3b,g, blue curves). In additiom, we show the measured and calculated amplitude part of the Husimi function, *Q*_meas_ and *Q*_BSV_, respectively (Extended Data Fig. 3b,g, grey curve). The mean intensity in the simulated shot-resolved spectra is 〈*I*_BSV_〉 = 1.5 × 10^12^ W cm^−2^.

We choose this intensity so that the simulation maximum of *P* (blue curve) in Extended Data Fig. 3g matches that of the experimental data in Extended Data Fig. 3b, again using the calibration via *I*_clas_ for the experimental axis. This intensity deviates from the experimental one obtained above (〈*I*_BSV_〉 = 3.9 × 10^12^ W cm^−2^) probably because of the exact shape of the spectra and Coulomb effects. In simulation, we also find that the probability distribution *P* and in particular its maximum also reflect the interplay of the nonlinear electron yield and the fast-decaying Husimi function (Extended Data Fig. 2a). Likewise, the simulated correlation map (Extended Data Fig. 3f) shows a similar yield behaviour along the vertical axis and, importantly, the same correlation between post-selected photon number *N*_BSV_ and the width of the electron energy spectra as in the experiment.

Comparing the simulated and measured shot-resolved electron spectra presented in Extended Data Fig. 3a,f, we find that our single-electron theory cannot explain the broadening of the spectra towards energies below zero (plus the bias voltage), which we observe in the experiment (Extended Data Fig. 3a,e). Experimentally, we observe a comparable shift towards negative energies for coherent light pulses at similar intensities (Extended Data Fig. 1). We expect that Coulomb repulsion of several tens to hundreds of electrons within one light pulse leads to the observed negative energies^57^ (see also ref. ^58^). This strong repulsion washes out the peak at low energies below 10 eV visible in the simulated shot-averaged spectrum (Extended Data Fig. 3h) but does not affect higher energies notably^57^.

Last, we note that the broad range of photon number distribution in the experiment allows us to probe the emission dynamics with BSV over a large range of Keldysh parameters: intensities exceeding 2 × 10^13^ W cm^−2^ are equivalent to a Keldysh parameter of *γ* < 0.7, placing the photoemission deep in the tunnelling regime, while the lowest cut-off energies indicate a Keldysh parameter of *γ* > 2, in the multi-photon regime. Based on the agreement between the simulated and measured shot-resolved energy spectra, we conclude that, in both the multi-photon and tunnelling regimes, the incoherent sum of Husimi function-weighted coherent-light spectra explains our averaged electron spectra.

### Simulation of multi-electron effects

As discussed in the section ‘Shot-resolved strong-field spectra’ and Extended Data Fig. 1, we attribute the experimentally observed broadening toward negative energies with increasing intensities to Coulomb repulsion effects (Fig. 3a,b). To support this interpretation, we use a three-dimensional point-particle simulation to study classical trajectory effects including rescattering and Coulomb repulsion. We assume that electrons are randomly born on a sphere with a radius *r*_tip_ = 300 nm. The initial spatial coordinates are given by the projection of a two-dimensional Gaussian function with *σ* = 10 nm onto the sphere, while the electron birth times are given by the Yudin–Ivanov rate^59^. These electrons are then propagated in the optical near field around the tip apex. We use coherent laser pulses with a pulse duration of *τ* = 38 fs, similar to the BSV pulses used in the experiment. Moreover, we apply a static bias voltage of −300 V, like in the experiment. For the potential landscape, we use the model of a spherical capacitor with the outer electrode set to infinity. This model does not account for the influence of the shank to the potential landscape at the apex, which is present in the experiment. Therefore, we chose a larger tip radius of *r*_tip_ = 300 nm for this simulation compared with the TDSE one, resulting in a static electric field of −1 V nm^−1^ at the surface. More details of the implementation of the simulations can be found in refs. ^57,60^.

**Extended Data Fig. 4:**
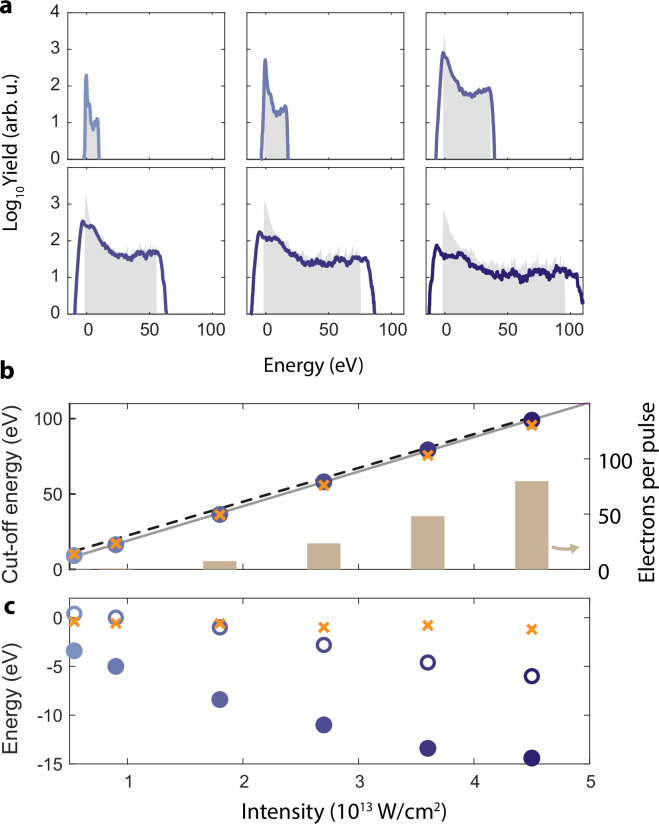
Simulation of multi-electrons effects. **a**, Energy spectra as a function of near field intensity without (grey area) and with Coulomb interaction (light blue to dark blue). The intensities are *I* = [0.54, 0.90, 1.8, 2.7, 3.6, 4.5] × 10^13^ W/cm^2^. We observe that Coulomb interaction can lead to slightly increased energies beyond the expected energy cut-off and, moreover, to negative energies, that is, energies below the energy resulting from the bias voltage applied to the tip. **b**, Cut-off energies (left-hand axis) with Coulomb interaction (circles; matching colors in **a**) and without Coulomb interaction (orange crosses). The right-hand axis shows the mean number of emitted electrons per pulse *μ*_*e*_ (brown bars, right-hand axis) as function of the near field intensity. Both with and without Coulomb interaction, the simulations show high agreement with the 10-*U*_p_-scaling law (black dashed line). The gray line is a linear fit for the case with Coulomb interaction, which yields a scaling of 10.2 × *U*_p_. **c**, Energy positions of the maximum of the energy spectra with (hollow circles) and without Coulomb interaction (orange crosses). The minimal energies with Coulomb interaction are shown as filled circles. We observe that for increasing intensity and, hence, with increasing *μ*_*e*_, both the minimum energies and the peak positions shift to negative energies. We observe energy shifts of ~ 15 eV. See text for details. Source data

In Extended Data Fig. 4a, we show the simulated energy spectra without Coulomb interaction (grey area) and with Coulomb interaction (light-blue to dark-blue curves) for six intensities *I* = [0.54, 0.90, 1.8, 2.7, 3.6, 4.5] × 10^13^ W cm^−2^. Here, we assume that the nonlinear emission yield scales with the near-field intensity according to a power law with an exponent of *n* = 3 (nonlinearity). The assumed mean numbers of emitted electrons per laser pulse *μ*_*e*_ are then *μ*_*e*_ = [0.10, 0.76, 7.5, 23.5, 48.0, 79.8] (Extended Data Fig. 4b, right-hand axis). The electron number from pulse to pulse fluctuates according to Poissonian statistics.

We observe that, for the three lowest intensities (upper row), Coulomb interaction has little effect on the high-energy electrons. Therefore, the highest energies that can be reached agree with the case without Coulomb interaction. For the three highest intensities, we observe electrons that exceed the energy expected from the 10-*U*_p_ law, visible as a soft edge (lower row). In this case, the Coulomb interaction of many electrons pushes a few electrons towards higher energies. We determine the cut-off positions of the spectra using two linear fit functions, similar to the method used in the experiment (Fig. 3b). The determined cut-off energies are shown in Extended Data Fig. 4b. We find that the resulting cut-off positions are almost the same with (light blue to dark blue) and without (orange) Coulomb interaction. Both cases are close to the expected 10-*U*_p_-cut-off scaling, which shows that Coulomb interaction does not have a large effect on the positions of the cut-off. By using a linear fit, we extract that the cut-off positions with Coulomb interaction scale as 10.2 × *U*_p_ (grey line) and in the case without Coulomb interaction as 9.5 × *U*_p_. The slightly reduced values without Coulomb interaction as compared with the 10-*U*_p_ cut-off are a result of the strong static electric field in the classical simulation^61^.

Previous experiments have shown that, when thousands of electrons per pulse are emitted at much higher intensities, strong deviations from the 10-*U*_p_ law can be expected, visible as a nonlinear behaviour of the cut-off energy as function of the intensity^62^. We do not observe this behaviour in our experiment (Fig. 3c).

Furthermore, our simulations show that as the intensity increases, the energies of the direct electrons become more smeared out (Extended Data Fig. 4a). Similar to the experiment, we observe energies below zero (that is, the energy resulting from the bias voltage applied to the tip). In Extended Data Fig. 4c, we show the peak positions of the simulated spectra. We observe that without Coulomb interaction (orange crosses) the maxima of the spectra are always close to zero energy. With Coulomb interaction (hollow circles), these maxima shift down to ~−6 eV for increasing intensity. Furthermore, we show the minimum energy of the spectra (filled circles) with energies down to ~−15 eV. This shift is a clear result of Coulomb interaction during the acceleration in the static potential, affecting low-energy electrons more heavily than fast, strongly driven electrons.

Therefore, most importantly, our multi-electron simulations demonstrate that, even with strong Coulomb interaction, the cut-off energy remains a valid marker for the optical near-field intensity. In our experiment, the shape and scaling of the plateau strongly suggest that it is caused by rescattering^42^, despite the presence of Coulomb repulsion effects (negative energies). Furthermore, our classical simulation results justify the applicability of the 10-*U*_p_ law and the use of the semi-classical TDSE simulation in one dimension, where an expansion to more dimensions (space and particles) is not possible due to computational reasons.

## Online content

Any methods, additional references, Nature Portfolio reporting summaries, source data, extended data, supplementary information, acknowledgements, peer review information; details of author contributions and competing interests; and statements of data and code availability are available at 10.1038/s41567-025-03087-1.

## Source data

Source Data Fig. 2 Line plots; experiment.

Source Data Fig. 3 Line plots and two-dimensional maps; experiment and theory.

Source Data Extended Data Fig. 1 Line plots; experiment.

Source Data Extended Data Fig. 2 Line plots; theory.

Source Data Extended Data Fig. 3 Line plots and two-dimensional maps; experiment and theory.

Source Data Extended Data Fig. 4 Line plots; simulation.

## Data Availability

All other data supporting the plots and findings presented in this Article are available from the corresponding author upon reasonable request. Source data are provided with this paper.
